# Encapsulation of Hydrophobic Phthalocyanine with Poly(*N*-isopropylacrylamide)/Lipid Composite Microspheres for Thermo-Responsive Release and Photodynamic Therapy

**DOI:** 10.3390/ma7053481

**Published:** 2014-04-30

**Authors:** Jiaojiao Liu, Jingliang Li, Zexin Zhang, Yuyan Weng, Gaojian Chen, Bing Yuan, Kai Yang, Yuqiang Ma

**Affiliations:** 1 Center for Soft Condensed Matter Physics and Interdisciplinary Research, Soochow University, Suzhou 215006, Jiangsu, China; E-Mails: 20114209069@suda.edu.cn (J.L.); zhangzx@suda.edu.cn (Z.Z.); wengyuyan@suda.edu.cn (Y.W.); gchen@suda.edu.cn (G.C.); yangkai@suda.edu.cn (K.Y.); 2 National Laboratory of Solid State Microstructures, Department of Physics, Nanjing University, Nanjing 210093, Jiangsu, China; 3 Institute for Frontier Materials, Deakin University, Waurn Ponds, Vic 3216, Australia; E-Mail: jingliang.li@deakin.edu.au

**Keywords:** phthalocyanine, phospholipid, composite microsphere, controlled release, photodynamic therapy

## Abstract

Phthalocyanine (Pc) is a type of promising sensitizer molecules for photodynamic therapy (PDT), but its hydrophobicity substantially prevents its applications. In this study, we efficiently encapsulate Pc into poly(*N*-isopropylacrylamide) (pNIPAM) microgel particles, without or with lipid decoration (*i.e*., Pc@pNIPAM or Pc@pNIPAM/lipid), to improve its water solubility and prevent aggregation in aqueous medium. The incorporation of lipid molecules significantly enhances the Pc loading efficiency of pNIPAM. These Pc@pNIPAM and Pc@pNIPAM/lipid composite microspheres show thermo-triggered release of Pc and/or lipid due to the phase transition of pNIPAM. Furthermore, in the *in vitro* experiments, these composite particles work as drug carriers for the hydrophobic Pc to be internalized into HeLa cells. After internalization, the particles show efficient fluorescent imaging and PDT effect. Our work demonstrates promising candidates in promoting the use of hydrophobic drugs including photosensitizers in tumor therapies.

## 1. Introduction

Phthalocyanines (Pc) and their derivatives have significant potential as theranostic agents for fluorescence imaging-guided drug delivery and photodynamic therapy (PDT) applications [1–3]. PDT is a kind of clinical therapeutic method during which the photosensitizers (e.g., Pc molecules) generate reactive oxygen species (ROS) upon absorption of light with specific wavelength, to kill tumor cells invasively or noninvasively [4,5]. Thus, during the photodynamic therapy using Pc molecules, following the accumulation of these photosensitizers in a tumor region under the visualizing and monitoring by fluorescence imaging, PDT can be precisely applied onto the targeted tissue by selectively illuminating the tumor cells, without hurting the normal organs around [2,5]. Furthermore, belonging to the second generation of sensitizer molecules used in PDT, Pc has strong absorption and fluorescence emission in the near-infrared (NIR) light range (called “biological optical window”), among which minimal tissue autofluorescence and light scattering occur [6,7].

However, the clinical application of Pc has been significantly limited due to its poor water solubility and tendency to form dimers in aqueous medium, which would affect their photophysical and photosensitizing properties and photodynamic action [8]. Various strategies have been explored to enhance the water solubility and overcome aggregation of Pc molecules, including chemical conjugation of them with hydrophilic polymers [9,10], and encapsulation of them into nano- or microcarriers, including micelles [11], liposomes [4], dendrimers [5,12], nanoparticles [13,14], *etc.* Among these formulas, noncovalent encapsulation of the hydrophobic Pc into carriers, such as macromoleculars and micro-/nanoparticles, is most well received due to the facile manipulation for drug encapsulation, enhanced drug loading efficiency, and preserved intrinsic properties of the carrier [5,7].

Microgel particles have emerged as a well received system for drug delivery [15–17]. These particles combine the unique properties of a gel with those of micro-/nanoparticles, such as improved drug loading capacity, tunable and monodispersed size, good aqueous dispersibility and high stability, as well as biocompatibility [15]. Poly(*N*-isopropylacrylamide) (pNIPAM) is a type of stimuli-sensitive polymer [18]. Besides the normal property of gelled particles, the pNIPAM microgel spheres demonstrate thermo-triggered volume phase transition behavior when crossing the lower critical solution temperature (LCST) of it at 32 °C [19]. That is, at a temperature below the LCST, the pNIPAM particle is in a swollen state with hydrated water molecules; however, when the temperature is above the LCST, the hydrogen bonding between the polymer and water molecules breaks down, much of the water is expelled from the inside of the particle leading to significant contraction (by ~40%) of the particle [20,21].

In one of our previous work we have reported that pNIPAM microgel particles with decorated lipids (namely “lipogel”) can be utilized as carriers of hydrophilic drugs, for controlled drug loading and release due to their biocompatibility and stimuli-responsive phase transition [20]. Now in this work, we successfully encapsulate hydrophobic Pc into pNIPAM microgel particles without or with lipid decoration. The as-prepared Pc@pNIPAM and Pc@pNIPAM/lipid composite microspheres show thermo-responsive drug release behavior and PDT effect in HeLa cells.

## 2. Results and Discussion

### 2.1. Characterization of pNIPAM, Pc@pNIPAM and Pc@pNIPAM/Lipid Microspheres

**Figure 1.**
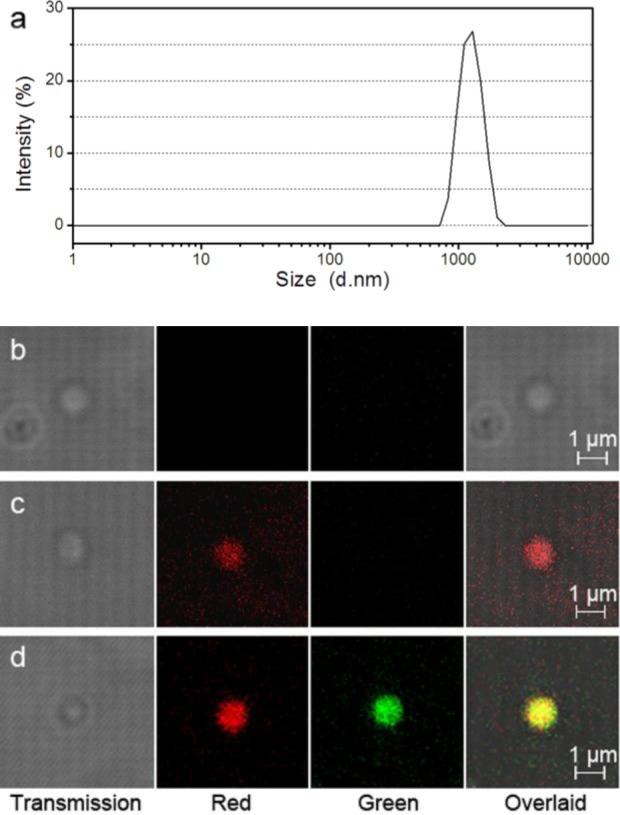
(**a**) DLS distribution of pristine pNIPAM microgel particles in aqueous dispersion; (**b**–**d**) confocal micrographs, including transmission, fluorescence, and overlaid images, of (**b**) pNIPAM; (**c**) Pc@pNIPAM and (**d**) Pc@pNIPAM/lipid microspheres in aqueous dispersions. Red, Pc; green, NBD-PE-labeled lipids.

The pristine pNIPAM microgel particles were 1.1 ± 0.1 μm in size (Figure 1a) and positively charged (*cf.* Supporting Information, Figure S1a). The size monodispersity of the particles was further confirmed by AFM experiments (Figure S1b). The three types of samples, *i.e*., pNIPAM, Pc@pNIPAM and Pc@pNIPAM/lipid microspheres, were observed on an inverted confocal microscope as shown in Figure 1b–d. The pristine pNIPAM microgel particles were invisible in the fluorescence channel while the Pc and lipid compositions of the composite microspheres were visualized in the red and green channels, respectively. Compared with the pristine pNIPAM microgel particles, the incorporation of Pc and lipids into the pNIPAM particles did not have much distinct impact on the size or morphology of the particles in optical or SEM observations (Figure S2). Except that, the Pc and lipid loaded particles became visualized under fluorescence observation, which endowed the particles with fluorescence imaging ability. The red and green fluorescence were distributed uniformly within the particles (Figure 1c,d).

**Figure 2.**
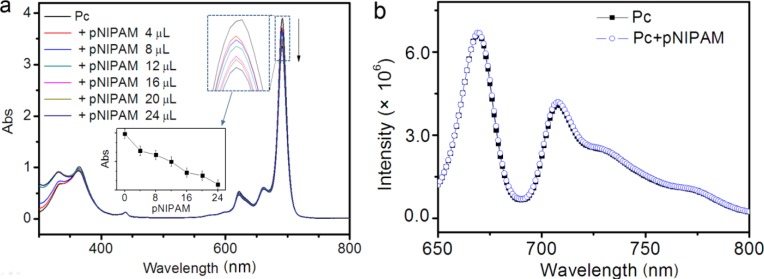
(**a**) UV–vis absorbance profiles of saturated Pc solution (in 3 mL THF) with the addition of increasing volumes of pNIPAM dispersion. Insets demonstrate the pNIPAM-quantity dependence of the intensity of the characteristic absorbance peak of Pc monomers at around 690 nm; (**b**) Fluorescence spectra of Pc solution before and after pNIPAM addition (pNIPAM = 15 µL, λ_exc_ = 630 nm). 1 μL pNIPAM dispersion contains ~10^9^ particles.

Figure 2a shows the Uv-vis absorbance spectra of Pc solution (saturated in 3 mL THF) upon the addition of different quantities of pNIPAM dispersion. Before each measurement, the solution was stabilized for half an hour after the *in situ* injection of pNIPAM. The absorbance peak at ~690 nm originates from the Pc monomers, while the broader peak at ~620 nm corresponds to Pc dimmers [22]. It is noted that Pc dimers are inactive and much more inefficient than monomers as photosensitizers for PDT [4,23]. With the addition of pNIPAM, the intensity of the absorbance peak referring to Pc monomers decreased in an approximately linear manner, without any disturbance to the other part of the profiles. This indicates that more and more Pc monomers are adsorbed into the pNIPAM particles, leading to the decrease of Pc concentration in the solution. On the other side, we found that after the addition of a certain amount of pNIPAM, the intensity of the absorbance peak decreased with time and did not reach an equilibrium, even after a long time of 3 h (not shown). This means that the adsorption of Pc into pNIPAM particles is a slow dynamic process. Here, the loading of hydrophobic Pc molecules into pNIPAM microgel spheres is facilely achieved by simple mixing of them. The incubation time dependence of Pc loading in pNIPAM particles will be further discussed later.

The steady-state fluorescence emission spectra of Pc without and with pNIPAM addition (also after being stabilized for 30 min) were analyzed as shown in Figure 2b. Both of them show two typical peaks at ~670 and ~710 nm, being associated with the loss of symmetry of Pc monomers in THF, and no distinguishable difference in fluorescence intensity was observed between the two systems [22]. This indicates that with the addition of pNIPAM, no much disturbance has occurred to the quantity or stabilization of the Pc monomers in the system. FT-IR spectra of these systems were also measured (Supporting Information, Figure S3). The characteristic peaks in Pc or pNIPAM were maintained in the Pc@pNIPAM composite and no new peaks were observed, indicating that no new covalent bond was formed in the composite. Thus, the loading of Pc into pNIPAM particles is probably due to the hydrophobic interactions between Pc and the nonpolar groups of pNIPAM polymers.

### 2.2. Incubation-Time and Lipid-Quantity Dependence of Pc Loading in the pNIPAM (with Lipid Decoration)Microspheres

**Figure 3.**
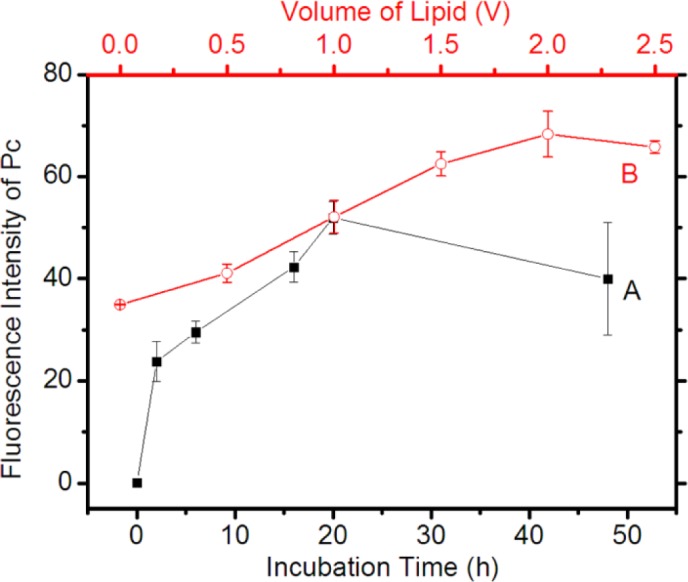
(**A**) Pc-incubation time dependence of the red fluorescence intensity (referring to the quantity of Pc loaded) of the as-prepared Pc@pNIPAM/lipid microspheres; (**B**) Fluorescence intensity distribution of Pc in the Pc@pNIPAM/lipid microspheres with different quantities of lipid during the composite preparation process. Here, 1 “V” corresponds to an amount of 0.2 mg lipid (see Experimental Section). The data of fluorescence intensity was obtained through integrating the fluorescence intensity of Pc in the Pc-loaded microspheres in the confocal micrographs acquired under the same equipment settings. An amount of 0.2 mg lipid and a Pc-incubation time of 20 h were generally used in the experiments unless stated otherwise.

As mentioned above, the adsorption of Pc into pNIPAM microgel particles, *i.e*., the formation of Pc@pNIPAM composite, is a time-dependent process, so is the Pc@pNIPAM/lipid system. We prolong the incubation time of Pc in the pNIPAM/lipid solution during the composite preparation process to a few hours and measure the quantity of Pc loaded in the composite as a function of the incubation time. Here, the changes in the quantity of Pc loaded in the Pc@pNIPAM/lipid composites are demonstrated through calculating the fluorescence intensity of Pc in model composite spheres in the confocal micrographs (an average of three model spheres was calculated in each experiment and four independently repeated experiments were performed). All these micrographs were acquired under the same equipment settings [20,24]. As shown in Figure 3A, within the first 20 h, the fluorescence intensity of Pc keeps on increasing with time. However, the increase in intensity is much faster within the initial 2 h than after that. This indicates fast adsorption of Pc at the initial stage followed by slow adsorption. No further increase occurred after a prolonged incubation time of 48 h, indicating the adsorption is saturated. On the basis of this information, in the following experiments, we chose an incubation time of 20 h for the Pc@pNIPAM/lipid composite preparation. On the other hand, we found that for the Pc loading in the pNIPAM/lipid composite system, the quantity of Pc loaded can be modulated through controlling the amount of lipid. Profile B in Figure 3 shows the lipid-quantity dependence of fluorescence intensity of Pc in the Pc@pNIPAM/lipid composite microspheres. In comparison with the pristine pNIPAM particles (*i.e*., lipid = 0 point), the addition of lipid significantly promotes the loading of Pc (about twice more). The quantity of Pc loaded increases with the amount of lipid in an approximately linear manner, until the lipid reaches 2.0 V (equals 0.4 mg). Such a linear dependence indicates that almost all the lipid has been incorporated into the composite microspheres. At the point of lipid = 2.0 V (*i.e*., lipid molecules:pNIPAM particles = 1.4 × 10^8^ by mol), the microspheres achieve a maximal loading capacity, after which no further increase in Pc quantity occurs. This indicates that the incorporated Pc has been saturated in the composite system.

Here, the addition of lipid significantly enhanced the physical encapsulation of hydrophobic drug Pc into pNIPAM microgel particles and resulted in a higher drug loading efficiency. The loading of Pc into the interior of pNIPAM microgel particles prevented aggregation of the encapsulated Pc in aqueous solutions and thereby preserved their photophysical properties required for efficient fluorescence imaging and PDT applications [5]. The remarkable efficiency of lipid to facilitate the loading of hydrophobic Pc into pNIPAM particles might be attributed to two reasons. Apart from the hydrophobic interactions between the large hydrophobic skeleton of Pc and the hydrophobic tails of lipid, the zwitterionic (both negatively and positively charged) head groups of lipids reduce the electrostatic repulsion between the identically charged Pc and pNIPAM particles, which also enhance the loading of Pc into the microspheres.

### 2.3. Thermo-Triggered Pc Release from the Pc@pNIPAM or Pc@pNIPAM/Lipid Microspheres

**Figure 4.**
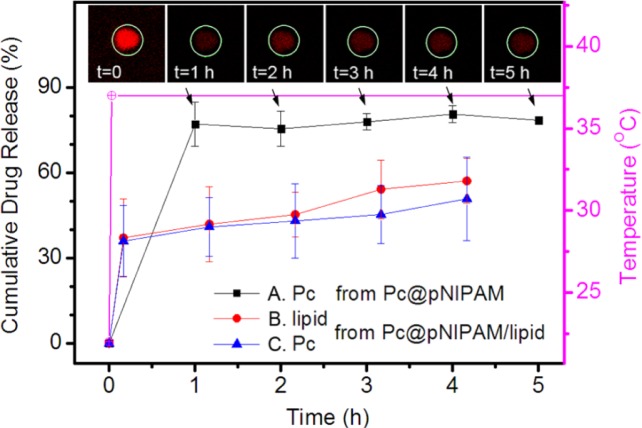
Release kinetics of Pc (and lipid) from the model Pc@pNIPAM and Pc@pNIPAM/lipid microspheres upon increasing the temperature from 22 to 37 °C. The release profiles were obtained through integrating the residual fluorescence intensity of Pc (or lipid) within the microsphere at certain time, as shown in the insets. The white circles in insets represent the area for integration of a model Pc@pNIPAM sphere while the red fluorescence originates from Pc. Averages were taken from four replicates.

To further explore the ability of the composite as an advanced drug carrier, the release of Pc (and lipids) from the composite at body temperature was investigated. Figure 4 show the temperature-triggered dynamic release process of Pc from the Pc@pNIPAM and Pc@pNIPAM/lipid systems. Noting that no much quenching of Pc or NBD occurs under similar experimental conditions (Supporting Information, Figure S4), the changes of red (or green) fluorescence intensity of the composite microsphere were employed to monitor the release of Pc (or lipid) from the composite. At a constant temperature of 22 °C, the fluorescence intensity of both the Pc@pNIPAM and the Pc@pNIPAM/lipid microspheres hardly changed, indicating that the incorporated Pc and lipids could be stably localized within the particles. For the Pc@pNIPAM system, when the temperature was increased from 22 to 37 °C, a burst decrease occurred to the fluorescence intensity of the composite (*i.e*., an increase in the cumulative Pc release from 0% to ~80%, Profile A in Figure 4). After that, the fluorescence intensity remained constant, even after a long time of 6 h. This means that the increase of temperature would trigger the release of most of the incorporated Pc from the inside of the particles, while the left Pc remains stable within the pNIPAM microspheres. In contrast, for the Pc@pNIPAM/lipid composite, a different release manner of Pc occurred. When the temperature was increased from 22 to 37 °C, the fluorescence intensity of Pc decreased with time quickly (corresponding to an increase in the cumulative Pc release from 0% to ~38%, Profile C in Figure 4). However, after that, the fluorescence intensity kept on decreasing in an approximately linear manner even after a long time of 4 h (to an ultimate Pc release of ~47%). Meanwhile, the changes in fluorescence intensity of the NBD-doped lipid in the composite microsphere were also monitored to learn about the dynamic process of lipid release from the composite. The release behavior of the lipid is similar to that of Pc (Profile B in Figure 4).

**Figure 5.**
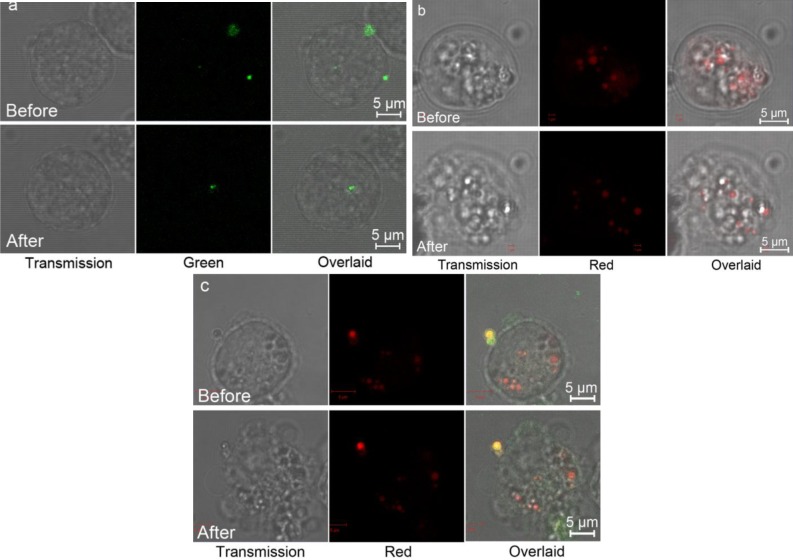
PDT effect of (**a**) pNIPAM/lipid; (**b**) Pc@pNIPAM; and (**c**) Pc@pNIPAM/lipid microspheres to HeLa cells, after light irradiation of 20 min. The confocal images of the cells (with the incorporated microspheres) before light irradiation were also shown for comparison. Red: Pc; green: NBD-labeled lipid; yellow in (**c**) overlaid of red and green.

It noted that when the temperature is increased from 22 to 37°C (crossing the LCST of the pNIPAM polymers), volume phase transition occurs to the pNIPAM microgel particles. During this phase transition process, the hydrogen bonds between the amide groups of polymer molecules and the surrounding water molecules break down and water is expelled from the vicinity of the polymer chains, leading to a significant volume contraction of the pNIPAM particle. The initial burst release of Pc and lipid in Figure 5 might result from such temperature-triggered volume contraction of the pNIPAM particle. After that, the lipid in the pNIPAM/lipid composite further decreases along with the release of Pc. Concerning that the incorporation of lipid in the pNIPAM particles facilitates the encapsulation of Pc within the particles, the further decrease of Pc might result from the release of lipid from the pNIPAM/lipid composite. An initial burst release followed by a sustained slow release provides the potential benefit to achieve improved therapeutic effect [25]. This indicates that the lipid-decorated pNIPAM sphere can function as a slow release vehicle for entrapped hydrophobic species at body temperature, in which the pNIPAM scaffold serves as a drug carrier and the lipids act to modulate the release of drug molecules.

Besides being an important component of cell membranes, it has been reported that lipid has a great diversity in molecular decoration which enables its functionalization for advanced biological applications including targeted drug release and gene therapy [26,27]. In this work, both Pc and lipid show stimuli-responsive release from the Pc@pNIPAM/lipid composite, indicating this composite can be potentially used as an advanced multi-drug carrier for both lipid and some hydrophobic drugs.

### 2.4. PDT Effects of Pc@pNIPAM and Pc@pNIPAM/Lipid Microspheres in HeLa Cells

The cellular uptake and PDT effect of the Pc@pNIPAM and Pc@pNIPAM/lipid microspheres were evaluated on HeLa cells. The pristine pNIPAM (with decorated lipid), Pc@pNIPAM and Pc@pNIPAM/lipid composite microspheres were incubated with HeLa cells at 37 °C for 2 h. Red (referring to Pc) and/or green (NBD-labeled lipid) fluorophores were found within the targeted cells (Figure 5, upper images in Figure 5a–c). This means that all the three types of particles are able to be internalized into HeLa cells [21]. Then, the PDT effects of these particles to the cells were checked after being irradiated by light for 20 min (Figure 5, bottom ones in Figure 5a–c). For the pNIPAM/lipid system, no change of the cellular morphology was observed. However, for both the Pc@pNIPAM and Pc@pNIPAM/lipid systems, significant morphological changes occurred to the targeted cells, indicating that the cells had been greatly destroyed. Singlet oxygen generated through the photosensitization process of Pc is believed to be the major cytotoxin responsible for the damage (Figure S5). Pc can generate ROS to injure cells upon laser activation without the need to be released from the carrier [5]. Furthermore, the intrinsic fluorescence properties of encapsulated Pc validate its role as an effective imaging agent for therapy or diagnosis applications.

## 3. Experimental Section

### 3.1. Materials

**Figure 6.**
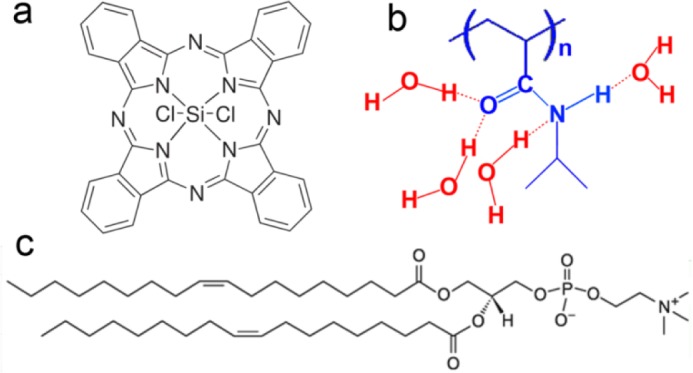
Chemical structure of (**a**) Pc; (**b**) pNIPAM (with hydrated water molecules); and (**c**) DOPC.

Silicon phthalocyanine dichloride (SiPcCl_2_, stated as Pc in this work) was bought from Sigma-Aldrich (St. Louis, MO, USA) and used as received (Figure 6a). The monomer *N*-isopropylacrylamide (NIPAM) was obtained from Acros (Geel, Belgium). 1,2-Dioleoyl-*sn*-glycero-3-phosphocholine (DOPC, Figure 6c) and 1,2-dioleoyl-*sn*-glycero-3-phosphoethanolamine-*N*-(7-nitro-2-1,3-benzoxadiazol-4-yl) (NBD-PE) were purchased from Avanti Polar Lipids (Alabaster, AL, USA). Chloroform (99.7%), tetrahydrofuran (THF, 99.0%), ethanol (99.0%) and the other chemicals used in the experiment (analytical reagent) were purchased from Shanghai Chemical Reagents Company (Shanghai, China) and used without further purification. Distilled water (>18 MΩ·cm) was produced using a Millipore filter system (Billerica, MA, USA). HeLa cells were cultured in Dulbecco’s Modified Eagle Medium (DMEM) at 37 °C and equilibrated in 4% CO_2_ and air.

### 3.2. Fabrication of pNIPAM, Pc@pNIPAM, and Pc@pNIPAM/lipid Microspheres

The pNIPAM microgel particles were synthesized using a semi-batch precipitation polymerization method, as reported in reference [28] and described in the supporting information (φ ~ 0.5, Section S2).

For the Pc@pNIPAM composite, a volume of 5 μL pNIPAM dispersion in water (containing about 3.6 ×10^9^ pNIPAM particles) was added to 1.5 mL saturated Pc solution in THF. The mixture was stirred for 20 min and dried under N_2_ flow. The product was rehydrated with 100 μL distilled water and vigorously stirred for dispersion. The obtained suspension was then centrifuged at 6000 rpm for 12 min. The precipitates (including Pc@pNIPAM composite) were re-suspended in 100 μL distilled water for use.

The Pc@pNIPAM/lipid composite was fabricated via a facile solvent-exchange method [29]. An amount of 0.2 mg lipid (DOPC labeled by 1 mol% NBD-PE, green fluorescence) was first dissolved in chloroform (2.0 mg mL^−1^) and dried overnight under vacuum. The dry lipid film was rehydrated with a 100 μL mixture of 40 vol% ethanol and 60 vol% Pc/pNIPAM mixed solution in water (prepared as mentioned above, before centrifuging). A volume of 1 mL distilled water was then added to the mixture. The bulk solution was centrifuged at 6000 rpm for 12 min. The wash and centrifugation were repeated three times. The precipitates (containing Pc@pNIPAM/lipid composite) were also re-suspended in 100 μL distilled water for use.

### 3.3. Characterization

UV-vis absorbance spectrum was collected with a SHIMADZU UV3600 spectrometer (Kyoto, Japan). Fluorescence measurements were performed with a HORIBA Jobin Yvon FluoroMax-4 Fluorescence Spectrometer (Paris, France). The zeta potential and size distribution of pNIPAM particles was determined using a Zeta Potential Analyzer (Zetasizer Nano ZS90, Malvern Instruments Ltd., Worcestershire, UK). All these tests were carried out at room temperature of 22 °C. Morphology of the microspheres was characterized on scanning electron microscope (Raith Pioneer, Dortmund, Germany, and HITACHI SU8010, Tokyo, Japan) after being freeze-dried.

The optical observation was performed on an inverted confocal fluorescence microscope (Carl Zeiss, LSM 710, Jena, Germany) equipped with an oil immersion objective (100×). Pc was excited at 543 nm and its fluorescence was collected in the red channel. The NBD-PE labeled lipid was excited at 488 nm and observed in the green channel.

### 3.4. Drug Release Test

The Pc-loaded microsphere (*i.e*., Pc@pNIPAM and Pc@pNIPAM/lipid) suspensions were transferred to a homemade sample cell for Pc release test under optical microscopic observation. The temperature of the system was set and stabilized with the native temperature control components from Zeiss. Throughout the release period, the settings including the laser power and amplifier offset were maintained constant [20,24]. The fluorescence intensity of Pc integrated from a model Pc-loaded particle was used to calculate the cumulative Pc release percentages, as: Cumulative Pc release at certain time (%) = (1−fluorescence intensity integrated from the Pc-loaded particle at certain time/fluorescence intensity at initial state) × 100%. The same method was used for the lipid release statistics [20].

### 3.5. Interactions between Particles and Cells and the PDT Effect Test

A volume of 500 μL buffer solution (including HeLa cells) was transferred to a homemade chamber equipped with the cell cultivation systems from Zeiss. After stabilization for 5 min (~4 cells per mm^2^ on the chamber substrate), 100 μL of pNIPAM/lipid, Pc@pNIPAM, or Pc@pNIPAM/lipid particle dispersion was injected slowly. The following interactions between particles and cells were monitored *in situ* with the confocal microscope. To test the PDT effect of the Pc-loaded composite microspheres to HeLa cells, a 633 nm laser at 2% power intensity was irradiated on the microsphere under monitoring with an exposure area of 20 × 20 μm^2^ for 20 min before observations.

## 4. Conclusions

We demonstrated that a model hydrophobic theranostic agent, Pc, can be efficiently encapsulated into pNIPAM microgel particles, without or with lipid decoration, for near-infrared photodynamic therapy (PDT) of cancer *in vitro*. The addition of lipid enhanced the loading efficiency of Pc in the pNIPAM particles. The Pc-loaded composite microspheres dispersed stably in aqueous solution. Temperature-triggered volume phase transition of pNIPAM led to a significant release of Pc (~80%) from the Pc@pNIPAM microspheres. However, for the Pc@pNIPAM/lipid composite, an initial burst release followed by a sustained slow release of both Pc and lipid occurred instead. Both the Pc@pNIPAM and Pc@pNIPAM/lipid composite spheres can be encapsulated by HeLa cells. Upon light irradiation, the cells were significantly destroyed due to the PDT effect of Pc. Such pNIPAM/lipid system promises applications as carriers for other hydrophobic drugs for imaging, diagnose and treatment of diseases.

## Supplementary Materials

Supplementary File 1
