# Supplementary material for: Encapsulation of Hydrophobic Phthalocyanine with Poly(N-isopropylacrylamide)/Lipid Composite Microspheres for Thermo-Responsive Release and Photodynamic Therapy
Source: Materials (Basel). 2014 Apr 30;7(5):3481–93. doi: 10.3390/ma7053481 (PMC5453233; doi:10.3390/ma7053481)
Supplement: Supplementary File 1 [file materials-07-03481-s001.pdf]

Supporting Information

S1. Supporting Figures

Figure S1. (**a**) zeta potential distribution of pNIPAM microgel particles in aqueous dispersion; (**b**) AFM image of the pNIPAM particles deposited on a silicon surface (measured in air phase). The height profile was measured along the red line showing the diameter of the particles.

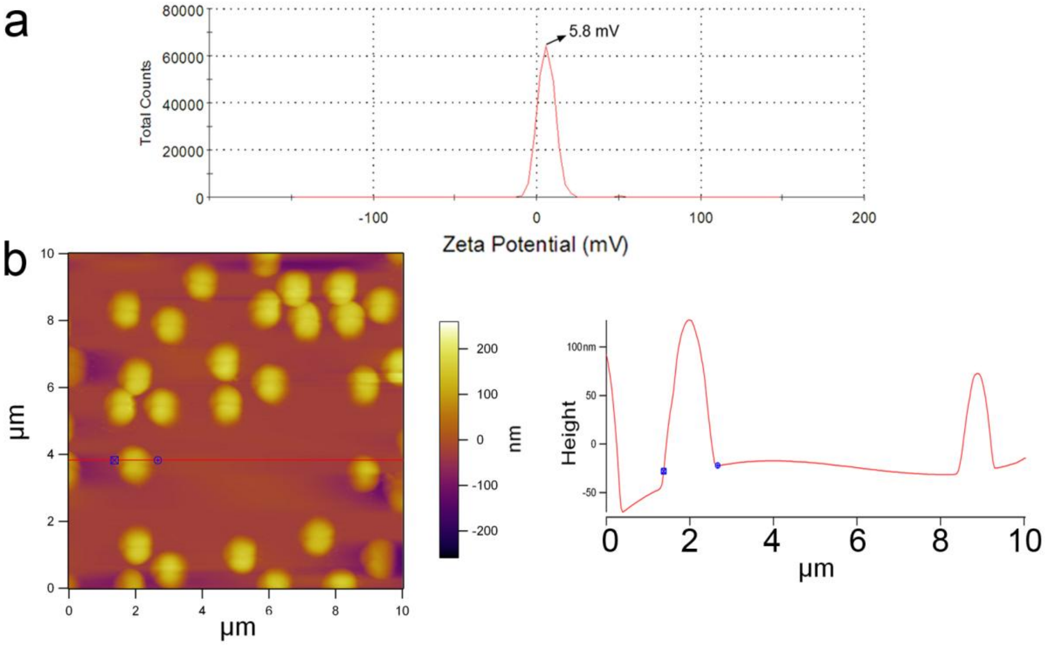

Figure S2. SEM images of model freeze-dried samples of (**a**) pNIPAM; (**b**) Pc@pNIPAM; and (**c**) Pc@pNIPAM/lipid microspheres; (**d**) confocal image of Pc@pNIPAM/lipid composite microspheres. White circles point out one typical sample shown in different channels.

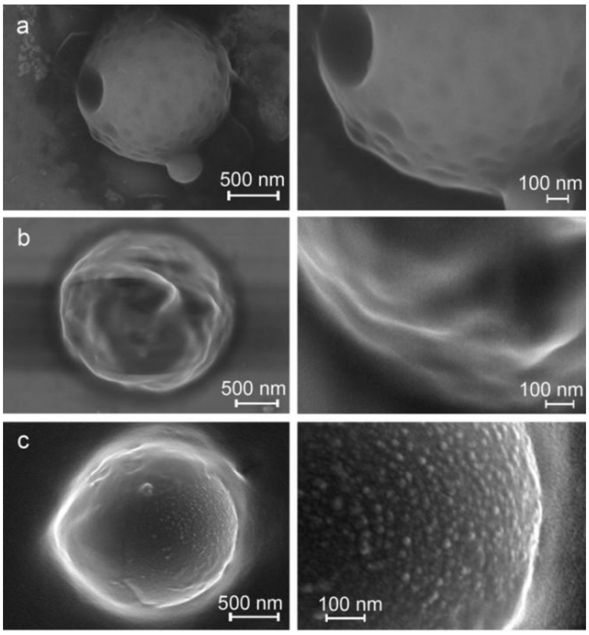

Figure S2. *Cont.*

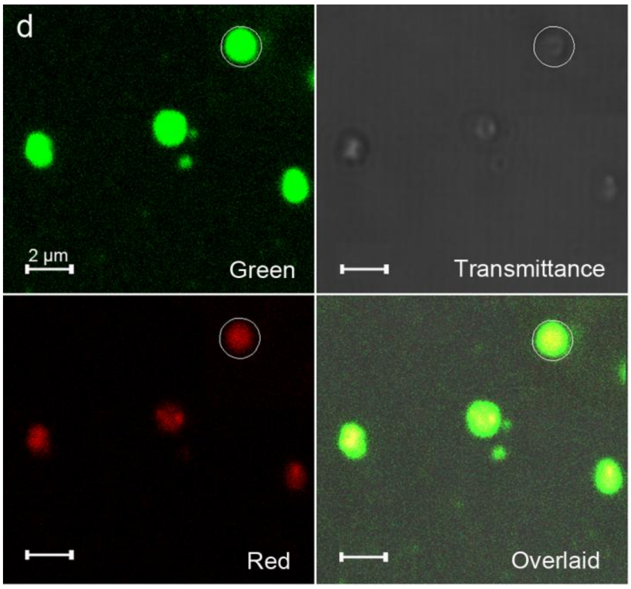

Figure S2a–c shows the SEM image of three types of samples, *i.e.*, pristine pNIPAM, Pc@pNIPAM and Pc@pNIPAM/lipid microspheres, after being freeze-dried. The pristine pNIPAM particles show spherical structure with porous and pitted surface due to the freeze drying treatment of the hydrogel particles before SEM observation. The Pc@pNIPAM composite spheres demonstrate a similar morphology of that of the pristine pNIPAM particles and maintain most of the holes or pits on the surface. In contrast, for the Pc@pNIPAM/lipid microspheres, the holes or pits look indistinct. Instead, the surface is densely covered with a large number of granular pimples. The increased pimples might refer to the freeze-dried lipid assemblies decorated on the surface of the composite spheres [1].

Figure S3. FTIR spectra of pNIPAM, Pc and Pc+pNIPAM.

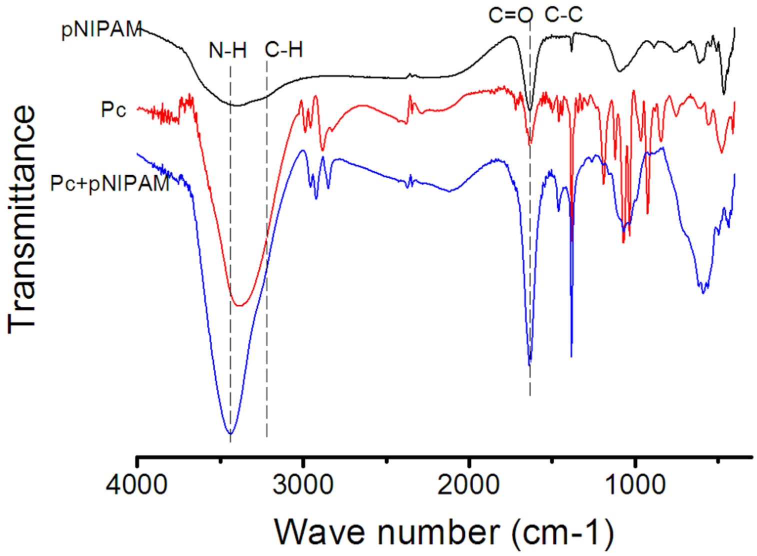

The FTIR spectrum of pNIPAM exhibited a broad peak of N-H stretching vibration at 3500 cm^{-1}, a peak of C-H at 3200 cm^{-1}, and a peak of the C=O stretching vibration at 1630 cm^{-1}. In the spectrum of Pc, the peak of the N-H stretching vibration at 3375 cm^{-1} was observed. Besides this, C-H and C-C of the aromatic ring at around 3300 and 1600 cm^{-1}, was observed respectively [2]. All these characteristic peaks were maintained for the Pc+pNIPAM hybrid system, while no new peak was observed.

Figure S4. Fluorescence quenching effect of NBD-labeled lipid with time. The initial fluorescence intensity was normalized as 1.

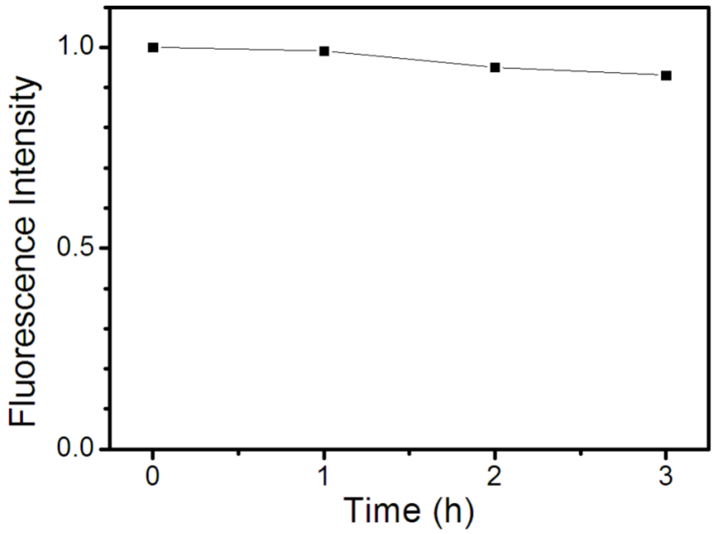

The fluorescence quenching of NBD-labeled lipid was tested with NBD-labeled lipid vesicles under similar experimental conditions at 22 and 37 °C, respectively. Figure S4 shows that only around 7% of the fluorescence was quenched after an incubation time of 3 h at 37 °C.

Figure S5. Control sample of HeLa cell with internalized Pc@pNIPAM/lipid microspheres without laser irradiation. No change of the cellular morphology was observed compared with those after light irradiation (*i.e.*, Figure 5c in the main text).

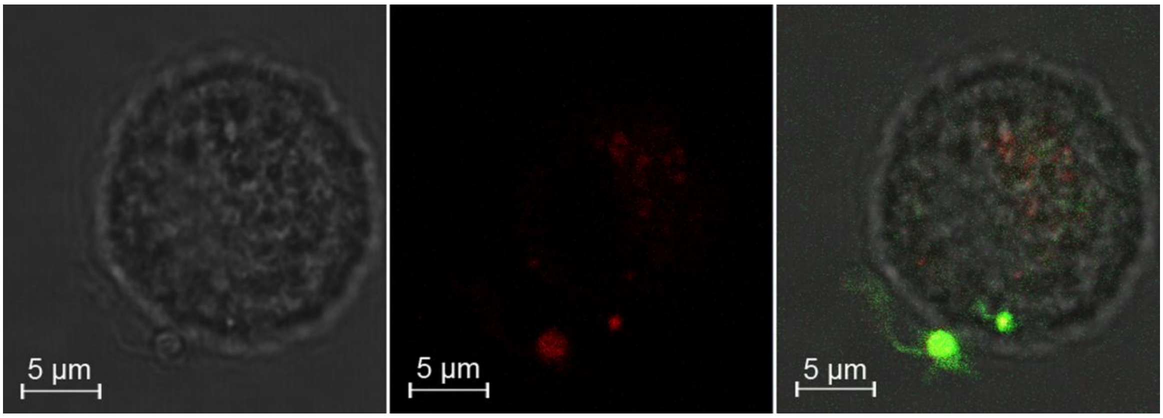

S2. Complementary Materials

Synthesis of pNIPAM Microgel Particles [3]

An amount of 0.9 g NIPAM monomer was dissolved in 55 mL distilled water and filtered to remove any solid impurities. The solution was purged with nitrogen gas to remove the dissolved oxygen, and stirred in a 40 ℃ water bath for 1 h. Then, 0.055 g KPS (in 1 mL distilled water) was added to the reaction vessel to initiate the polymerization. Two minutes later, 6 mL solution containing 0.3 g NIPAM, 0.032 g MBA, and 0.15 mL MAA was added at a rate of 5 mL per hour. When the reaction mixture started to turn opalescent, the temperature was ramped to 60 °C in 1 h. After all of the 6 mL comonomer solution was added, the reaction mixture was stirred for 2 h more at 60 °C.

References

1. Lu, N.; Yang, K.; Li, J.; Weng, Y.; Yuan, B.; Ma, Y. Controlled drug loading and release of a stimuli-responsive lipogel consisting of poly(N-isopropylacrylamide) particles and lipids. *J. Phys. Chem. B* **2013**, *117*, 9677–9682.
2. Dong, J.; Weng, J.; Dai, L. The effect of graphene on the lower critical solution temperature of poly(*N*-isopropylacrylamide). *Carbon* **2013**, *52*, 326–336.
3. Kwok, M.-H.; Li, Z.; Ngai, T. Controlling the synthesis and characterization of micrometer-sized PNIPAM microgels with tailored morphologies. *Langmuir* **2013**, *29*, 9581–9591.
